# ‘Will polar bears melt?’ A qualitative analysis of children’s questions about climate change

**DOI:** 10.1177/0963662520952999

**Published:** 2020-09-04

**Authors:** Katharine Lee, Julie Barnett

**Affiliations:** University of Bath, UK

**Keywords:** children, climate change, psychological distance

## Abstract

Climate change poses a grave threat to future generations, yet relatively little research examines children’s understandings of the issue. This study examines the questions children ask about climate change – rather than their answers to adults’ questions – exploring whether their questions suggest they view climate change as psychologically proximal or distant. Children aged 10–12 from 14 UK schools took part in an online event, asking scientists questions in a ‘climate zone’. The questions were analysed using thematic analysis. The themes related to the nature and reality of climate change, its causes, impacts, and solutions. Participants seemed most exercised about the future impacts of and ways of ameliorating climate change, with some questions evoking science-fiction disaster imagery. The contents of participants’ questions elucidated the ways in which they position climate change as both a proximal and distant phenomenon.

## 1. Introduction

The nature of the potentially devastating impact of climate change on the planet and its occupants is now established (Masson-Delmotte et al., 2018). Parties to the United Nations Framework Convention on Climate Change (UNFCC) reached an agreement in 2016 (COP16) to pursue efforts to limit the global mean temperature increase to 1.5°C. To achieve this, action will need to be taken globally, with responsibility assumed by governments, corporations and individuals. The next generation of adults will inherit this responsibility as they become decision-makers and voters. They will need to be equipped to deal with the challenges brought by the future impacts of climate change. Given this, it is surprising that in the academic literature to date, more attention has been paid to the climate change related perceptions of adults than of children and adolescents. A better understanding of children and adolescents’ perceptions of climate change can help inform the way that climate change is communicated to children to best engender their current and future pro-social behaviour and tackle their anxieties in the most helpful way. It could also inform the content and timing of climate change teaching in the curricula.

One concept that has frequently been explored in relation to adults’ perceptions of climate change is psychological distance, that is, the propensity to locate the problem of climate change at a distance from the self along spatial, temporal, social and hypothetical lines (Trope and Liberman, 2010). Many scholars have argued that perceiving climate change as more proximal makes it more personally salient (e.g. Reser, Bradley, Ellul) and increases concern and willingness to engage with climate change (Jones et al., 2017). However, the link between a proximal climate change and engagement is not necessarily straightforward (e.g. Brügger et al., 2015).

Little is known about children and adolescents’ psychological distancing of climate change. There is some evidence that younger people are more likely to view climate change as psychologically distant than older adults (Corner et al., 2015). A 2019 study found that while Swiss adolescents viewed climate change as a real and current threat (hypothetically and temporally proximal), they saw it as a greater threat to other people in other places (socially and spatially distant; Gubler et al., 2019). Here, a link between psychological closeness and increased concern was established. A recent systematic review concluded that youth in the United Kingdom may view climate change as psychologically distant (Lee et al., 2020), although no study in the review addressed the concept specifically. In one international study about personal transport in the context of climate change, participants in the United Kingdom expressed the lowest level of concern (50%) of the 11 included countries, whereas participants in India (89%) expressed much higher levels of concern (Boyes et al., 2014). Participants in the United Kingdom were also far less willing – relative to the extent to which they agree the actions were useful – to take public transport or drive a smaller car, than those living in India. These differences in levels of concern and willingness to act may arise from participants in the United Kingdom viewing climate change as a greater threat to other countries; indeed, climate change currently presents a greater risk to India than the United Kingdom (Eckstein et al., 2019). The extent to which children and adolescents view climate change as psychologically distant or proximal, warrants further investigation.

Having established that psychological distance is a useful framing device for exploring children’s perceptions of climate change, the question of appropriate methods arises. Much of the research about adolescents’ perceptions of climate change takes a ‘knowledge-deficit’ perspective. That is, assessing the extent to which reported beliefs about climate change correspond to scientific ones (e.g. Punter et al., 2011), the difference being attributed to a shortfall in knowledge or information (Suldovsky, 2017). Other studies measure participants’ attitudes towards climate change and/or their willingness to take or support particular actions that will contribute to ameliorating its effects (e.g. Chhokar et al., 2012). Participants in these studies respond to a closed-form survey, where responses are usually measured on a five-point scale. Such studies require participants to answer a set of narrow questions which specify both the facets of climate change of interest and the register of permissible responses. Participants can reveal something about how much they know about the factors laid before them, or how willing they might be to take designated actions but nothing about their thoughts and understandings beyond these parameters. Findings from some open-response surveys and qualitative studies have indicated that enabling a broader set of responses leads to more nuanced understanding. For example, concern about climate change seems flexible and context-dependent in qualitative studies in China (Sternäng and Lundholm, 2011, 2012), when responses to closed-form questionnaires indicate very high concern (Boyes et al., 2008). This suggests that participants in closed-form survey studies may be prompted to express what appear to be higher levels of concern when response options are pre-specified and contextual factors omitted.

Many qualitative studies have used open-response surveys, interviews or focus groups (e.g. Scott-Parker and Kumar, 2018). Although these enable a greater breadth of responses, they preserve a hierarchical relationship, with adult researchers directing and child participants responding. In contrast, this study seeks to privilege the participant by enabling them to ask their own questions about climate change. This approach has been employed relatively infrequently, and mainly in the field of education, by researchers investigating children’s interests in science and technology (e.g. Baram-Tsabari and Yarden, 2005), students’ interest in chemistry (Demirdogen and Cakmakci, 2014) and young adults’ interest in climate change (Tolppanen and Aksela, 2018).

With participants asking the questions, control over the direction of enquiry is placed with them (Ripberger, 2011). Question asking is – or has the potential to be – predicated on a desire to know the answer to that particular question, rather than on the requirement to provide an appropriate answer to another’s question (Demirdogen and Cakmakci, 2014). Questions reveal what participants want to know about a particular object (Chin and Osborne, 2008), what they may already think and feel about it (Baram-Tsabari et al., 2006), and the nature of their expectations of science (Falchetti et al., 2007). In analysing their questions, we do not seek to compare the accuracy of children’s knowledge with scientific knowledge, or to measure the extent to which they express certain attitudes or beliefs, rather to understand their sense-making around the issue.

This study takes place in the United Kingdom, where – contrary to practice elsewhere, where it is taught earlier (George, 2017) – formal teaching about climate change occurs in the second or third year of secondary school (Department for Education, 2014). The study is conducted with participants aged 10–12 in the United Kingdom who are pupils in years 6 and 7 at school, the last year of primary education and the first year of secondary. While their teachers may have chosen to incorporate climate change into their lesson plans, these children have not reached the stage of receiving formal teaching on climate change in the Geography and Science curricula.

The research questions are the following:

1. What questions do 10- to 12–year-old children ask about climate change?
2. Is psychological distancing of climate change evident in these questions?
3. If so, how is this psychological distancing of climate change expressed?

## 2. Method

### Design

The study generated qualitative data within a cross-sectional research design. The study received ethical approval from the University of Bath in January 2018.

### Procedure

‘I’m a Scientist, get me out of here!’ is an online event organised by a company whose focus is engaging schoolchildren with tertiary science educators. Teachers sign up to take part and each class is allocated a ‘chat’ session that runs during a lesson. During the event, students interact with up to six scientists in science-themed ‘zones’, asking them questions relating to the zone topic and more general questions. Zones relate to a broad range of scientific topics (e.g. Stress, Food, Gravity). Some zones are designed for primary school-aged children, some for secondary school-aged children, others for both. Participants are given non-identifying usernames. Each session features at least one and often several scientists. Chats are moderated to ensure that questions remain civil. Participants may ask additional questions through a separate ‘Ask’ section at any time during the event. Scientists reply to these questions outside the session. The ‘Climate zone’ ran for 2 weeks in March 2018. Six scientists took part in the event. Three were PhD students, in earth sciences, social science, and environmental microbiology. The others were a climate and environmental specialist, renewable energy manager, and climate data scientist.

### Participants

Seven primary schools (13-year six classes) and seven secondary schools (12-year seven classes) took part in the event. Children were aged between 10 and 12. Ten of the schools were in England, two in Scotland, one in Wales and one in Northern Ireland. Most of the schools were mixed sex. Ranked by index of multiple deprivation (National Statistics, 2015; Scottish Government, 2016; StatsWales, 2014), the English schools’ postcodes were located between the 10% and 20% most deprived neighbourhoods in the country (two schools), and the 10% least deprived (one school), with the remaining schools spread between these poles. In Scotland, the two schools were in the 30% most deprived and 30% least deprived, respectively. The Welsh school was in one of the 30% most deprived postcodes. Data were not available for Northern Ireland.

### Analysis

The analysis was informed by a deductive–inductive hybrid framework (Fereday and Muir-Cochrane, 2006). This approach integrates a deductive analysis informed by the four types of psychological distancing (Trope and Liberman, 2010), with an inductive, data-driven analysis. The questions relating to climate change were analysed using thematic analysis (Braun and Clarke, 2006), in order to identify meaningful patterns in the data. The authors did not seek to code with a focus on constructing reliable categories as we were working within a qualitative approach that took ‘an organic approach to coding and theme development’(Clarke and Braun, 2018: 108), frequently discussing the material.

Exactly duplicated questions were removed. The remaining questions were read through several times, then coded according to subject matter. Observations about the way in which questions were asked (e.g. language use or points of emphasis) were noted. Finally, themes and sub-themes were identified. For consistency, question marks have been added to all questions and first words capitalised. Other grammatical or spelling errors have not been corrected.

## 3. Results

**Figure 1.**
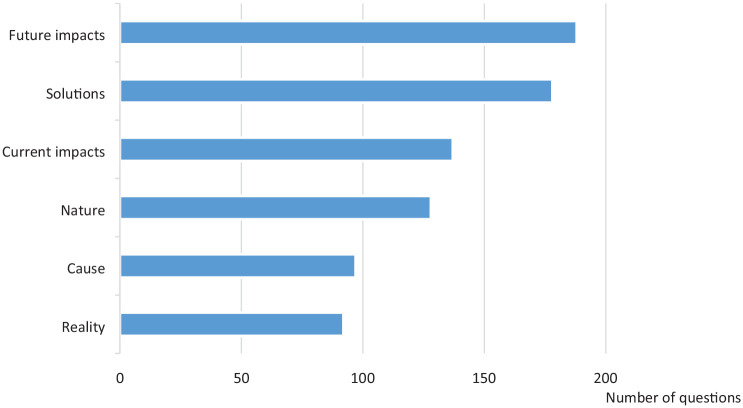
Number of questions in each category.

Of the 10,100 lines in the Chat and Ask sections, there were 820 unique questions relating specifically to climate change. Other questions that did not relate to climate change were excluded (e.g. General Certificate of Secondary Education (GCSEs) needed for a science career, the daily routine of a scientist, food and entertainment preferences). We identified the following six categories of questions about climate change: ‘*The nature of climate change*’, ‘*The causes of climate change*’, ‘*The current impacts of climate change*’, ‘*The future impacts of climate change*’, ‘*Resolving climate change*’ and ‘*The reality/severity of climate change*’. The distribution of questions is shown in Figure 1. A description of each category and sub-themes with exemplar questions are detailed below.

## 4. The nature of climate change

The questions in this theme comprised requests for information about the essential attributes of climate change. There were the following two sub-themes:

### What is climate change?

Questions here tended to be broad and open. In explicitly asking the scientists to explain the concept of climate change, participants acknowledged that establishing the nature of the phenomenon is a legitimate and necessary line of enquiry.

### What is the timeframe?

These questions related to establishing a timeline, past, present and future. The way that some questions were phrased seemed to indicate climate change being framed as an ‘event’ that would occur, or had occurred, rather than an ongoing issue.

**Table:** 

Sub-theme	Exemplar questions – the nature of climate change
What is climate change?	- How could you simply explain climate change? - Do you know why climate change is very important? - What is the difference between weather and climate change?
What is the timeframe?	- When did the climates start to change? - When will climate change be at its worst? - When will climate change end?

## 5. The causes of climate change

The questions within this category related to causes of climate change. The following two sub-themes were identified:

### What are the causes?

Some questions were open, others related to existing ideas about causes, such as pollutants or gases. Few questions alluded to actions that they, or ordinary people, might take.

### Who or what causes climate change?

Questions here related to countries and pollutants. They did not typically refer to individual behaviours.

**Table:** 

Sub-theme	Exemplar questions – the causes of climate change
What are the causes?	- What impacts climate? - Why are fossil fuels so damaging even though they are made out of natural resources? - Does littering affect climate change?
Who or what causes climate change?	- Which countries contribute the most to climate change or global warming? - Which gas affects the climate the most? So the worst greenhouse gas?

## 6. The current impacts of climate change

Questions in this category were written in the present tense. There were the following two sub-themes:

### What are the impacts?

Some questions were broad, but many related to specific and differentiated impacts. This indicates that these participants have a sense that a range of current activities and practices are impacted by climate change. However, impacts were generally geographically removed from the United Kingdom.

### Where are the impacts happening?

These questions related to the geographical location of climate change impacts. Almost all questions seemed predicated on the assumption that impacts are affecting places other than the United Kingdom.

**Table:** 

Sub-theme	Exemplar questions – the current impacts of climate change
What are the impacts?	- What are the worst effects of climate change? - Are animals such as polar bears who live in cold climates endangered? - Does climate change have something to do with spreading diseases?
Where are the impacts happening?	- What is the main country that has been affected by climate change? - What country is most affected by climate change? - Where does climate change affect the world the most?

## 7. The future impacts of climate change

Questions in this category were written in the future tense. The imagined future consequence ranged from the mundane to the apocalyptic. The following three sub-themes were identified.

### What are the future impacts?

These questions tended to be specific, relating to future impacts affecting animals, farming, food and humans. Many questions invoked science-fiction tropes. Participants appeared to align some of the future consequences of climate change with disasters depicted in films or video games.

### Where will the future impacts happen?

Questions here referred to the geographical location of future impacts and few related to the United Kingdom. The language around the geographical location of future impacts seemed predicated on greater certainty around the more distant impacts; questions relating to the United Kingdom used more speculative language.

### When will the future impacts happen?

Questions in the final sub-theme aimed to establish a timeline to future events. Some questions specified timeframes and were fatalistic in tone. Some questions related to participants’ own lifetimes and to specific consequences. Some alluded to completely catastrophic consequences.

**Table:** 

Sub-theme	Exemplar questions – the future impacts of climate change
What are the future impacts?	- Will polar bears melt? - What happens if the world goes over 4oC? - What do you think will happen when the sun explodes? - Can climate change blow up the earth or aliens instead? - Do you think we will run out of air?
Where will the future impacts happen?	- Will climate change affect everybody or just a couple of countries? - Which countries will have the deadliest temperatures? - Can global warming affect Britain?
When will the future impacts happen?	- How long before global warming kills us all? - How long will it be until all cities are submerged through climate change? - When will the earth explode? - Could humans and living things become extinct in the next century?

## 8. Resolving climate change

Questions in this category related to potential solutions to climate change. Three sub-themes were identified.

### Can it be stopped?

These questions seemed underpinned by uncertainty about whether anything could be done, some questions implied that it might already be too late.

### What can be done?

The personal pronouns employed in the questions in this sub-theme indicated which agents were positioned as responsible or able to provide solutions to climate change. First person pronouns such as ‘I’, or more frequently ‘we’, indicated a focus on what could be done individually or collectively. Second person pronouns indicated that responsibility was placed with others, often scientists. Some questions indicated a misapplication of evolutionary concepts, with the notion that humans might rapidly evolve or adapt to avoid the future impacts.

### Science-fiction solutions?

In the final sub-theme, participants invoked science-fiction solutions. They enquired about evacuating Earth and colonising other planets or the moon.

**Table:** 

Sub-theme	Exemplar questions – resolving climate change
Can it be stopped?	- Is there a way to avoid climate change? - Can the process be slowed down/stopped in any way? - Is it too late to do anything about it?
What can be done?	- What is the main thing that as humans we can do to slow down climate change? - Have you been trying to stop global warming? - What technology do you think will help solve global warming? - Should we be vegetarians then? - Will climate change make us evolve to adapt to constant increase in heat, snow and rain?
Science-fiction solutions	- Can we live on Mars to not get the affect of climate change PLEASE ANSWER thank you? - If we had to evacuate Earth what planet would you say we would have to move to and why? - When the world ends, could we be able to begin life on the moon? - What year do you think we will have to evacuate?

## 9. The reality/severity of climate change

The questions in this category expressed doubt about the veracity or severity of climate change. Three sub-themes were identified.

### Is climate change real?

These questions asked whether climate change exists, with some participants requesting proof. Some of the questions related to the colder-than-usual weather at the time of data collection, implying that this may mean that climate change is not happening.

### Is climate change caused by human activity?

Questions here related to non-human causes of climate change. These questions seemed predicated on acknowledgement that climate change is real, but uncertainty about its human cause. Again, participants sometimes asked the scientists to provide proof.

### Is it that bad?

These questions sought positives to set against the acknowledged negatives of climate change.

**Table:** 

Sub-theme	Exemplar questions – The reality/severity of climate change
Is climate change real?	- Is global warming complete hogwash? - How can scientists prove global warming is real? - Is your research accurate? - Why did we get more snow if the earth is getting warmer?
Is climate change caused by human activity?	- Is the climate change a natural process that the planet goes through every so many thousands or millions of years? - Are human activities or natural variations in climate responsible for climate change being observed today? - What proof do we have that climate change is being caused by humans?
Is it that bad?	- Could climate change be good in any way? - Can climate change somehow be beneficial towards us? - Although climate change is bad, are there any positive points about it?

## 10. Discussion

### What questions were asked?

We analysed the questions that 10- to 12-year-olds in the United Kingdom asked scientists about climate change. Their questions related to a range of issues, from the nature and reality of the phenomena, to its causes, impacts and solutions. The questions evidenced a broad awareness of many of the current and future impacts of climate change and their potential seriousness. Questions relating to solutions revealed a focus on what could be done – individually or collectively – with responsibility also placed with scientists and technology. Allusions to science-fiction were made around future impacts and solutions. Some of the questions expressed scepticism about climate change. While the content of some questions could be interpreted to reveal a shortfall in scientifically accurate knowledge, they also reveal the ways in which a focus only on knowledge does not account for the rich and varied ways that children think about climate change. For example, it is notable that participants did not seem to make what might seem intuitive links between causes and impacts of and solutions to climate change. Most questions relating to solutions to climate change did not refer to the concepts mentioned in questions about causes. This suggests that they may not see them as sides of the same coin but as discrete issues.

### Is climate change viewed as psychologically distant?

Their questions indicate that participants see climate change as psychologically distant and proximal. The following four dimensions are discussed in turn:

#### Temporal distance

The evidence around temporal distancing was mixed. Questions about the nature and future impacts of climate change seeking to establish the timeline of climate change indicate that some participants may not be sure whether climate change is temporally proximal or distant. While the focus on future impacts is suggestive of temporal distancing, many questions indicated that participants view climate change as a current threat.

#### Spatial distance

Questions relating to current and future climate change impacts referred much more to the distant than local, indicating that climate change is spatially distant. This was underlined by use of speculative language in relation to impacts in the United Kingdom, and more certain claims around impacts elsewhere.

#### Social distance

The questions were indicative of climate change being socially proximal and distant. Questioners were often socially distant from climate change impacts, but also asked about its impact on ‘us’. The use of personal pronouns suggests that some consider themselves responsible for resolving climate change – albeit more collectively than individually – although scientists and technology were also described as able to provide solutions.

#### Hypothetical distance

Questions relating to scepticism demonstrated that for some participants, climate change is hypothetically distant, although many questions intimated certainty that climate change is a real phenomenon. Some questions contained explicit scepticism about climate change and askers did not couch their questions in a way they might indicate they felt the topic was taboo. We identified the following three types of scepticism: relating to whether the climate is really changing, whether changes are human-caused and whether it is ‘all’ bad. This mirrors the framework of Rahmstorf (2004).

#### Associations with science-fiction

It could be that associating climate change with science-fiction is a means of psychological distancing insofar as aligning it with something so inherently abstract makes it more distant. This could perhaps fit within social or temporal distancing, in that science-fiction is typically removed from the self and set in the future, although participants sometimes placed themselves within the science-fiction scenarios, by referring to ‘we’ and ‘us’. The more dramatic apocalyptic outcomes and science-fiction solutions seem the product of participants’ imagination mediating their understanding of risk (Yusoff and Gabrys, 2011), imaginings borrowed from scenes in science-fiction films or video games. Although science and science-fiction analogies are associated (Hughes et al., 2008), previous research with young participants has not, to our knowledge, revealed such direct associations with science-fiction. There is likely a methodological explanation for this; quantitative methods commonly used in previous studies do not present children and adolescents with apocalyptic outcomes and science-fiction solutions, but rather with scientifically viable potential impacts and solutions (e.g. Boyes et al., 2014; Daniel et al., 2004).

### Strengths, limitations and future research

The key strength of this study is the method, which has enabled us to access participants’ thoughts and ideas about climate change, some of which would likely not have been expressed in response to researchers’ questions. In asking their own questions, participants directed the enquiry, highlighting to us what areas are of interest to them. Their questions are informative, both in relation to providing answers to the questions that researchers would perhaps ask, but importantly in providing some answers to questions we would not think to ask. They also highlighted the contexts in which the concept of psychological distance is expressed.

The environment in which the children participated had arguably greater ecological validity than a survey study, although we do not know the extent to which children may have felt some pressure to perform as they would in class, because of their teacher’s presence.

The findings indicate avenues for future research. First, further exploration is warranted of the nuanced ways in which psychological distancing of climate change is manifested in the views of adolescents. Building on this, it is important to establish how this relates to expressions of concern and the kinds of pro-environmental behaviours – individual and collective – that young people are empowered to carry out, rather than hypothetical future behaviour (Ambusaidi et al., 2012). These data were collected in Spring 2018, prior to the publication of the most recent IPCC report (IPCC, 2018) and the ‘school strike for climate’ protests, so it would also be interesting to examine whether and how these events impact youth discourses around climate change and the questions they ask about it. The relationship between psychological distancing and participation in the strikes could also usefully be explored.

## 11. Implications

The findings here may have implications for young peoples’ willingness to behave environmentally and their well-being. Viewing climate change as a distant problem may reduce both its salience and propensity for engagement. Distancing, as well as the focus on devastating outcomes, may serve to obscure the arguably more likely – but rarely mentioned – impacts on people in the United Kingdom, such as water shortages and flooding (Environment Agency, 2018). Viewing science – and science-fiction – as the provider of solutions to climate change may be disempowering in that it could diminish the need for action at all levels, as well as creating a false sense of security. Framing climate change as completely disastrous could lead to disillusionment and apathy (Ojala, 2015) rather than hope and engagement (Ojala, 2012). This raises questions about what and when children should be taught about climate change in school. Some of the science-fiction ideas evidenced in the questions indicate that these participants are generating intuitive but inaccurate theories about the science of climate change (Carey, 2009), which may persist if they are not addressed (Kelemen and Rosset, 2009). Some of the questions relating to future impacts were fearful in tone. Fear may be a helpful tool to encourage climate-friendly action (Witte and Allen, 2000), but needs to be coupled with a sense that something can be done (Smith and Leiserowitz, 2014) in order to avoid feelings of hopelessness, a sense of which can increase across childhood (Ojala, 2013). It seems sensible to consider the benefits of introducing climate change into the curriculum at an earlier age, with a focus on dispelling potentially fear-inducing myths, scepticism, and making the issue more salient – and less psychologically distant – by focusing on the local and personally relevant.
